# Exogenous pyruvate facilitates cancer cell adaptation to hypoxia by serving as an oxygen surrogate

**DOI:** 10.18632/oncotarget.10202

**Published:** 2016-06-21

**Authors:** Chengqian Yin, Dan He, Shuyang Chen, Xiaoling Tan, Nianli Sang

**Affiliations:** ^1^ Department of Biology, Drexel University College of Arts and Sciences, Philadelphia, Pennsylvania, USA; ^2^ Department of High Altitude Physiology, the Third Military Medical University, Chongqing, China; ^3^ Department of Pathology and Laboratory Medicine, Drexel University College of Medicine, Philadelphia, Pennsylvania, USA; ^4^ Department of Dermatology, Boston University School of Medicine, Boston, Massachusetts, USA

**Keywords:** ATP, hypoxia, metabolism, pyruvate, NAD^+^

## Abstract

Molecular oxygen is the final electron acceptor in cellular metabolism but cancer cells often become adaptive to hypoxia, which promotes resistance to chemotherapy and radiation. The reduction of endogenous glycolytic pyruvate to lactate is known as an adaptive strategy for hypoxic cells. Whether exogenous pyruvate is required for hypoxic cell proliferation by either serving as an electron acceptor or a biosynthetic substrate remains unclear. By using both hypoxic and ρ^0^ cells defective in electron transfer chain, we show that exogenous pyruvate is required to sustain proliferation of both cancer and non-cancer cells that cannot utilize oxygen. Particularly, we show that absence of pyruvate led to glycolysis inhibition and AMPK activation along with decreased NAD^+^ levels in ρ^0^ cells; and exogenous pyruvate increases lactate yield, elevates NAD^+^/NADH ratio and suppresses AMPK activation. Knockdown of lactate dehydrogenase significantly inhibits the rescuing effects of exogenous pyruvate. In contrast, none of pyruvate-derived metabolites tested (including acetyl-CoA, α-ketoglutarate, succinate and alanine) can replace pyruvate in supporting ρ^0^ cell proliferation. Knockdown of pyruvate carboxylase, pyruvate dehydrogenase and citrate synthase do not impair exogenous pyruvate to rescue ρ^0^ cells. Importantly, we show that exogenous pyruvate relieves ATP insufficiency and mTOR inhibition and promotes proliferation of hypoxic cells, and that well-oxygenated cells release pyruvate, providing a potential *in vivo* source of pyruvate. Taken together, our data support a novel pyruvate cycle model in which oxygenated cells release pyruvate for hypoxic cells as an oxygen surrogate. The pyruvate cycle may be targeted as a new therapy of hypoxic cancers.

## INTRODUCTION

Cell survival, proliferation and other cellular functions require constitutive supply of molecular oxygen and nutrients to maintain ATP production, redox homeostasis, and biosynthesis [1]. However, cells in ischemic lesions and solid tumors commonly suffer from hypoxia [2]. Hypoxia not only impairs mitochondrial respiration and decreases ATP production, but also causes accumulation of NADH along with NAD^+^ depletion [3]. It has also been reported that hypoxia leads to formation of excessive reactive oxygen species (ROS), particularly superoxide, causing oxidative stress [4, 5].

One of the best known adaptive mechanisms of hypoxic cells, the Pasteur effect, is mainly induced by hypoxia-inducible factors (HIFs). Particularly, HIF-1 transcriptionally reprograms glucose metabolism, shifting ATP production from oxygen-dependent oxidative phosphorylation to NAD^+^-dependent glycolysis [6]. Hypoxia activates HIF-1 functions and transcriptionally upregulates the expression of glucose transporters, glycolytic enzymes and regulatory proteins of glycolysis. The elevated glycolysis maintains ATP levels in hypoxic cells, facilitating cell survival and other functions in the hypoxic microenvironment [7–9]. HIF-1-stimulated expression of lactate dehydrogenases (LDH) accelerates the conversion of endogenous pyruvate generated from glycolysis to lactate, recovering NAD^+^ by accepting electrons from NADH, thus maintaining a continuous glycolysis without complete NAD^+^ exhaustion [10].

In addition to hypoxia, defect in electron transport chain (ETC) blocks oxygen utilization in oxidative phosphorylation. Since a functional ETC depends on 13 proteins encoded by the mitochondrial genome [11], cells depleted of mitochondrial DNA (ρ^0^ cells) cannot use molecular oxygen as the final electron acceptor for cellular respiration, providing a unique model to study cellular adaptation to defective oxygen utilization [12]. Interestingly, the ETC-defective ρ^0^ cells absolutely depend on exogenously supplemented pyruvate for survival and proliferation [13], indicating that exogenous pyruvate, in addition to pyruvate endogenously generated from glycolysis, plays a critical role in cells that cannot use molecular oxygen.

Pyruvate is a critical node in multiple metabolic pathways for both biosynthesis and NAD^+^ homeostasis [14, 15]. However, the precise role of exogenous pyruvate in cell adaptation to hypoxia remains unclear. In this study, using both cancer and non-cancer cells, we demonstrate that endogenous pyruvate from glycolysis is insufficient to support continuous ATP production in cells with severe deficiency in oxygen utilization. Importantly, exogenous pyruvate is required to support the survival and proliferation of hypoxic cells mainly by acting as an oxygen surrogate to accept electrons, thus maintaining NAD^+^ homeostasis and ATP levels. The biosynthetic role of pyruvate as a carbon source is generally dispensable in cell proliferation. Furthermore, we show that well-oxygenated cells release pyruvate. Our data suggest a novel pyruvate cycle model that well-oxygenated cells secrete pyruvate to support the survival and proliferation of hypoxic cells, which uptake exogenous pyruvate as an oxygen surrogate in redox metabolism, thus facilitating ATP homeostasis and hypoxic adaptation.

## RESULTS

### Exogenous pyruvate is absolutely required for the proliferation of ETC-defective cells

**Figure 1:**
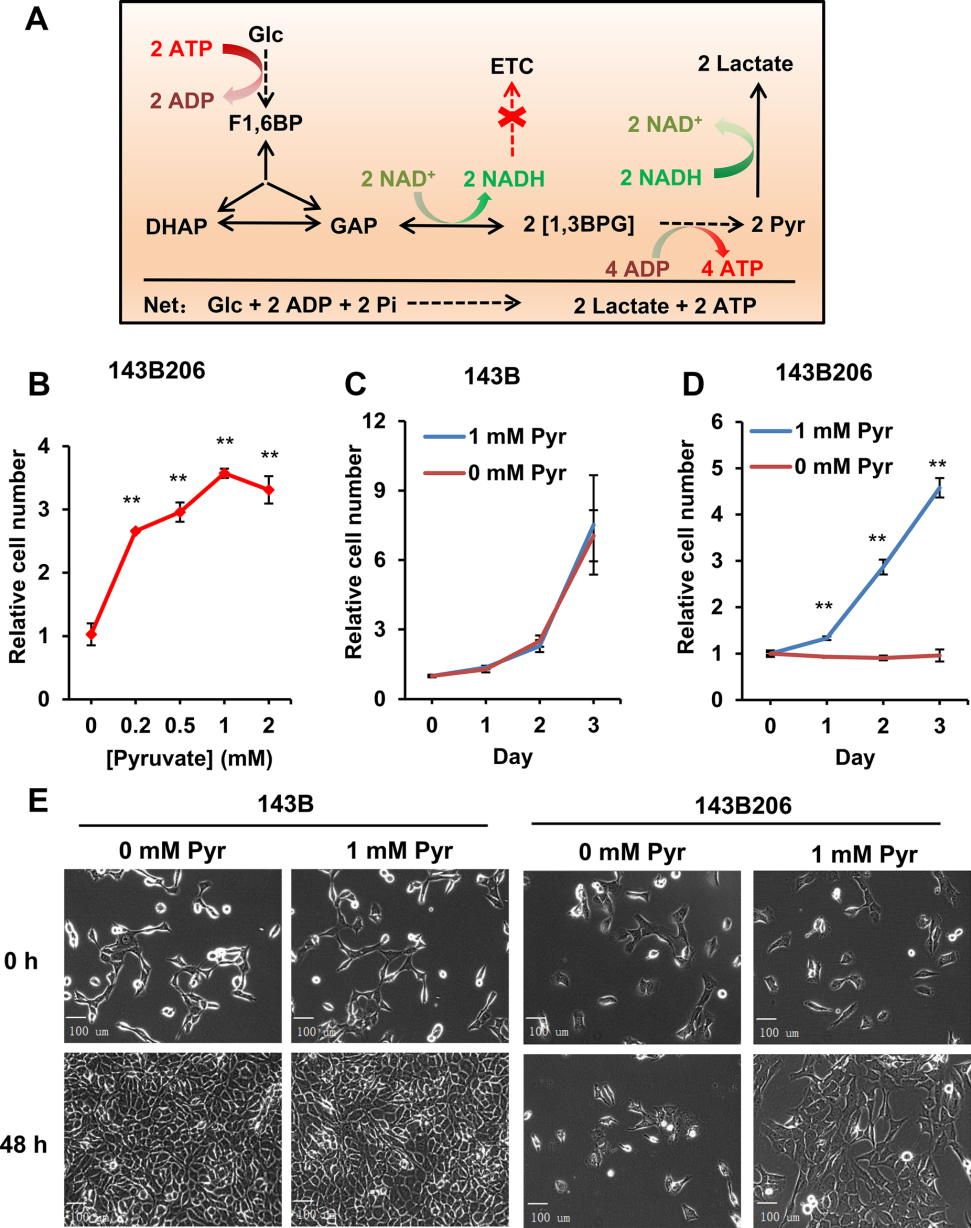
Endogenous pyruvate from glycolysis is not sufficient to support 143B206 proliferation (**A**) Stoichiometric summary of glycolysis and NAD^+^ homeostasis in 143B206 cells. (**B**) 143B206 cells were seeded in 96-well microplates and cultured overnight, then the media with indicated concentrations of pyruvate were used to culture cells for additional 48 h. Cell numbers were measured using CyQUANT assay. The relative cell numbers were normalized with the initial number of plated cells. (**C** and **D**) Proliferation rates of 143B206 and the parental 143B cells in the presence or absence of 1 mM pyruvate for indicated time were measured. (**E**) Morphology of 143B and 143B206 cells cultured in the presence or absence of 1 mM pyruvate was observed with Zeiss Axioplan microscope at 400× magnification. Bars represent 100 μm. Error bars indicate SD of ≥ 5 replicates. **p < 0.01.

To investigate the biochemical roles of exogenous pyruvate in cellular adaptation to defective utilization of oxygen, we exploited the metabolic properties of the ρ^0^ 143B206 cell line [13]. The glycolysis and pyruvate metabolism in this cell line were outlined in Figure 1A. The 143B206 cell line is derived from 143B through chronic exposure to ethidium bromide (EtBr), which disrupted the replication of mitochondrial genome [13]. Mitochondrial genome encodes 13 proteins that are components of Complex I, III, IV and V in ETC [11]. Due to lack of mitochondrial DNA, 143B206 cells have no functional ETC and, like anoxic cells, exclusively rely on glycolysis and lactate fermentation for ATP production and homeostasis of NAD^+^/NADH [16]. For glycolysis of one glucose molecule, two molecules of NAD^+^ need to be reduced to 2 molecules of NADH, which consume two molecules of pyruvate generated endogenously from glycolysis in lactate fermentation (Figure 1A). Therefore, based on stoichiometry all endogenous pyruvate generated from glycolysis in 143B206 cells would be converted to lactate in order to maintain the NAD^+^ homeostasis, simplifying the analysis of the role of exogenous pyruvate.

Firstly, we investigated the effects of increasing concentrations of exogenous pyruvate on the proliferation of 143B206 cells. Without exogenous pyruvate, the cell number increase of 143B206 cells was inhibited, which is consistent with previous reports [13]. Addition of pyruvate, as low as 0.2 mM, was sufficient to promote the proliferation of 143B206 cells significantly, and 1 mM pyruvate showed the best effect (Figure 1B). Under normoxic conditions (21% O_2_) exogenous pyruvate did not affect the proliferation of 143B (Figure 1C). However, ETC-defective 143B206 cells failed to proliferate in pyruvate-free media, which was rescued by addition of 1 mM exogenous pyruvate (Figure 1D). We also determined the morphological change of 143B206 cells with light microscopy. After 48 h of pyruvate withdrawal there was no visible morphological change in 143B206 cells, but cell proliferation was inhibited. As a control, the parental 143B cells maintained persistent and rapid proliferation regardless of the addition of exogenous pyruvate (Figure 1E). These data confirmed the dependence of 143B206 on exogenous pyruvate for proliferation.

### Exogenous pyruvate acts as the electron acceptor in ETC-defective cells

**Figure 2:**
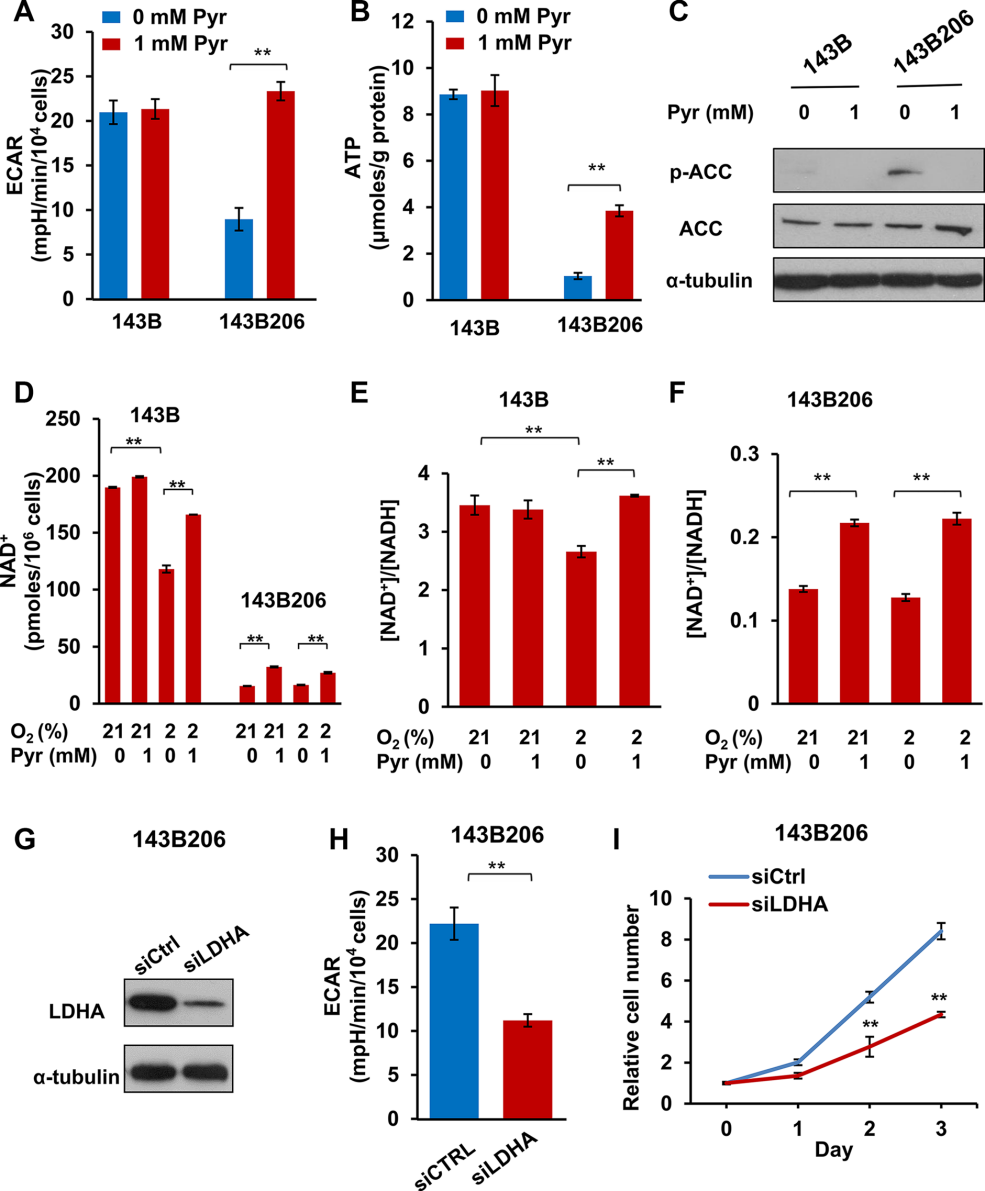
Exogenous pyruvate relieves NAD^+^ depletion and glycolysis inhibition (**A**) The average extracellular acidification rates (ECAR) were analyzed with Seahorse analyzer in 143B and 143B206 cells with 1 mM pyruvate or not. Error bars indicate SD from 4 replicates. (**B**) 143B206 and 143B cells were cultured with indicated conditions for 24 h. Cellular ATP levels were measured and normalized by protein content. Error bars indicate SD from triplicates. (**C**) Phosphorylated ACC (Ser79) and total ACC in whole lysates of 143B and 143B206 cells were analyzed with immunoblotting. α-tubulin was used as loading control. Representative result from triplicates is shown. (**D**) NAD^+^ concentrations in the lysates of 143B and 143B206 cells were measured and used to calculate the total amount of NAD^+^ in both cell lines (per 10^6^ cells). (**E** and **F**) NAD^+^/NADH ratio in 143B and 143B206 cells was calculated and presented. Error bars indicate SD of triplicates. (**G**–**I**) 143B206 cells were cultured in media with 1 mM of pyruvate and 50 μg/mL uridine. The knockdown efficiency of LDHA with siRNA was analyzed with immunoblotting and shown in (G) The effects of LDHA knockdown on ECAR were shown in (H) The effects of LDHA knockdown on 143B206 proliferation were shown in (I) Error bars indicate SD of triplicates. **p < 0.01.

The well-known role of pyruvate in Pasteur and Warburg effect in cancer metabolism is to accept electrons from NADH, being reduced to lactate by LDH. We asked whether the critical role of exogenous pyruvate in supporting 143B206 proliferation is to maintain NAD^+^ homeostasis. We first utilized the Seahorse metabolic analyzer to measure extracellular acidification rate (ECAR), the indicator of lactate formation. The parental 143B cells cultured with 21% O_2_ maintained an ECAR at 21 ± 1.32 mpH/min/10^4^ cells, which was not affected by the addition of exogenous pyruvate (Figure 2A). In contrast, the ETC-defective 143B206 cells showed an ECAR value at 9 ± 1.27 mpH/min/10^4^ cells in the absence of exogenous pyruvate, indicating that glycolysis was inhibited, instead of being stimulated. Importantly, addition of exogenous pyruvate dramatically increased the ECAR to 23 ± 1.04 mpH/min/10^4^ cells (Figure 2A), indicating that exogenous pyruvate promotes lactate generation. We also compared the oxygen utilization in 143B and 143B206 cells in the presence or absence of pyruvate. 143B cells showed typical OCR profiles similar to other cancer cells. 143B206 cells had no significant amount of oxygen consumption, which was not affected by the presence of exogenous pyruvate, further confirming that ETC is deficient in 143B206 cells (Supplementary Figure S1A–S1D).

Considering ETC-defective 143B206 cells showed inhibited glycolysis, we next investigated the effect of exogenous pyruvate on ATP production. ATP levels in 143B and 143B206 cells cultured under normoxic condition were measured. We observed that exogenous pyruvate had no obvious effect on the ATP level in 143B cells (which is around 9.0 μmoles/g protein), but the ATP level in 143B206 cells with absence of exogenous pyruvate was very low (1.04 ± 0.14 μmoles/g protein), which was significantly increased by exogenous pyruvate to 3.85 ± 0.24 μmoles/g protein (Figure 2B). AMP-activated protein kinase (AMPK) senses AMP levels and is activated upon ATP depletion [17]. One of the best known direct targets of AMPK is acetyl-CoA carboxylase (ACC), which is inhibited by AMPK-catalyzed phosphorylation at Ser79, thus being widely used as a cellular marker for ATP insufficiency [18]. Under normoxic conditions, phosphorylated ACC was not detectable in 143B cells but was easily detected in 143B206 cells (Figure 2C). As we predicted, addition of exogenous pyruvate significantly decreased the level of phosphorylated ACC (Ser79) without altering the total levels of ACC (Figure 2C).

A major direct metabolic consequence of lactate generation is the oxidation of NADH to NAD^+^ [19]. The low ECAR, decreased ATP levels and activation of AMPK in 143B206 cells indicate a depletion of NAD^+^. To investigate the effect of exogenous pyruvate on intracellular NAD^+^ levels and NAD^+^/NADH ratios, we cultured 143B and 143B206 cells under conditions specified in Figure 2D–2F. The parental 143B cells had an NAD^+^ level of 189.80 ± 0.03 pmoles/10^6^ cells. Hypoxia decreased NAD^+^ level to 118.06 ± 0.16 pmoles/10^6^ cells which was restored by exogenous pyruvate (Figure 2D). In comparison, the ETC-defective 143B206 had a dramatically lower NAD^+^ level at 15.39 ± 0.01 pmoles/10^6^ cells, and addition of exogenous pyruvate significantly elevated the NAD^+^ level to 32.21 ± 0.03 pmoles/10^6^ cells. We note that the pyruvate-elevated NAD^+^ levels remained lower than that of the parental cells. Consistently, 143B cells maintained NAD^+^/NADH ratio at 3.46 ± 0.17 under normoxia. Hypoxia decreased the NAD^+^/NADH ratio to 2.66 ± 0.10, which was prevented by the addition of exogenous pyruvate (Figure 2E). Since 143B206 cells have no functional ETC, the NAD^+^/NADH ratio maintained at a low level (0.14 ± 0.003), and the availability of oxygen showed no effect on that ratio (Figure 2F). However, addition of exogenous pyruvate significantly increased the NAD^+^/NADH ratios under both normoxic and hypoxic conditions (Figure 2F).

LDHA encodes one subunit of LDH, which can be induced by hypoxia, and has been considered as part of the cellular adaptive mechanism to hypoxia [20]. To further confirm the critical role of pyruvate as an oxygen surrogate, we knocked down LDHA in 143B206 cells and assayed the growth curve in the presence of 1 mM pyruvate. Transfection of LDHA siRNA in 143B206 cells significantly decreased the protein level of LDHA (Figure 2G) and led to a slowdown of ECAR (Figure 2H). Accompanied by the decrease of LDHA protein level and ECAR, the proliferation rate of 143B206 cells decreased significantly in the presence of pyruvate (Figure 2I).

Taken together, these data demonstrate that exogenous pyruvate rescues 143B206 cell by maintaining the homeostasis of intracellular NAD^+^, which is the determining factor of survival and proliferation of 143B206 cells. These data also imply that under severe hypoxia, exogenous pyruvate is absolutely needed to serve as an oxygen surrogate to maintain minimal NAD^+^ levels, thus ensuring the continuation of glycolysis and ATP production.

### Rescuing effects of pyruvate-derived metabolites on 143B206 proliferation

**Figure 3:**
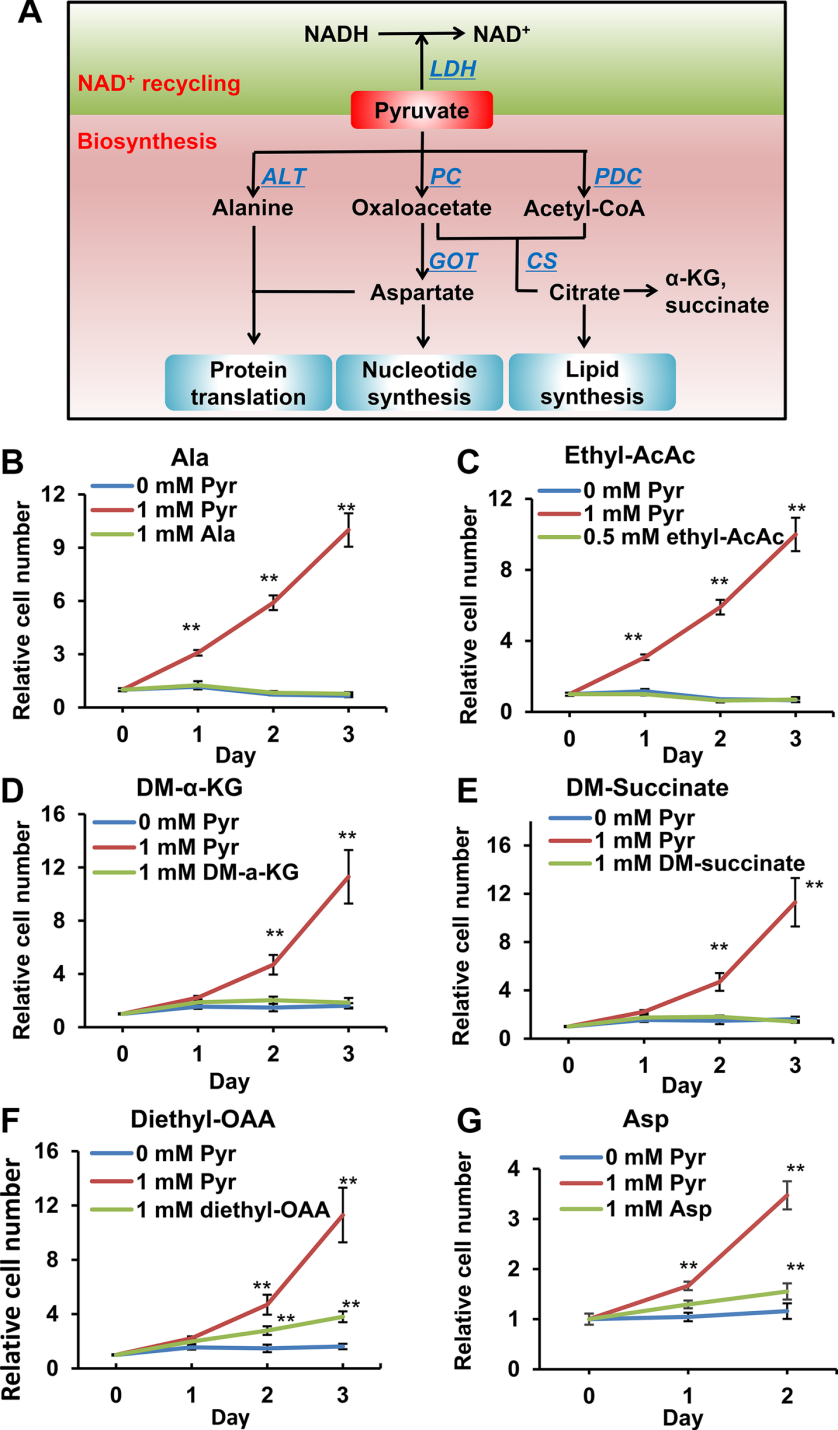
Effects of pyruvate-derived metabolites on 143B206 cell proliferation in the absence of pyruvate (**A**) Schematic summary of pyruvate-participated metabolic pathways. In addition to serving as electron acceptor, pyruvate may be used as a substrate in the synthesis of various metabolites, including acetyl-CoA, α-KG, succinate, OAA, alanine and aspartate. These metabolites can be used for the synthesis of proteins, lipids and nucleotides, thus supporting cell proliferation. (**B**) 1 mM alanine, (**C**) 0.5 mM ethyl-acetoacetate, (**D**) 1 mM dimethyl-α-KG, (**E**) 1 mM dimethyl-succinate, (**F**) 1 mM diethyl-OAA and (**G**) 1 mM aspartate were used to replace pyruvate to rescue 143B206 cells. Representative proliferation curves are shown. Error bars indicate SD of ≥ 5 replicates. **p < 0.01.

Pyruvate is at a pivotal node in cellular carbon metabolism, contributing carbon to the synthesis of various metabolites, including acetyl-CoA, α-ketoglutarate (α-KG), succinate, oxaloacetate (OAA), alanine (Ala) and aspartate (Asp) (Figure 3A). To determine if any of these pyruvate-derived metabolites are rate-limiting metabolites in 143B206 cell proliferation, we used each of these metabolites and examined if any metabolite can replace pyruvate to rescue 143B206 cell proliferation. Acetoacetate can be converted to acetyl-CoA by acetoacetate succinyl-CoA transferase and thiolase (Supplementary Figure S2A). Since acetoacetate, α-KG, succinate and OAA cannot diffuse across the cell membrane effectively, we exploited their modified precursors ethyl-acetoacetate, dimethyl-α-KG, dimethyl-succinate and diethyl-OAA. The alkyl groups can be hydrolyzed and removed by intracellular esterase after entering the cells [21–23]. We observed that the addition of Ala, acetyl-CoA, α-KG and succinate could not replace exogenous pyruvate to support 143B206 cell proliferation (Figure 3B–3E). OAA and Asp partially rescued the proliferation of 143B206 cells; however, their rescuing effects were significantly lower than exogenous pyruvate (Figure 3F and 3G), and their rescuing effect may be ascribed to the fact that OAA may serve as an electron acceptor in redox metabolism (Supplementary Figure S2B, S2C).

### Suppression of the biosynthesis of OAA, acetyl-CoA and citrate does not impair the rescuing effect of exogenous pyruvate

**Figure 4:**
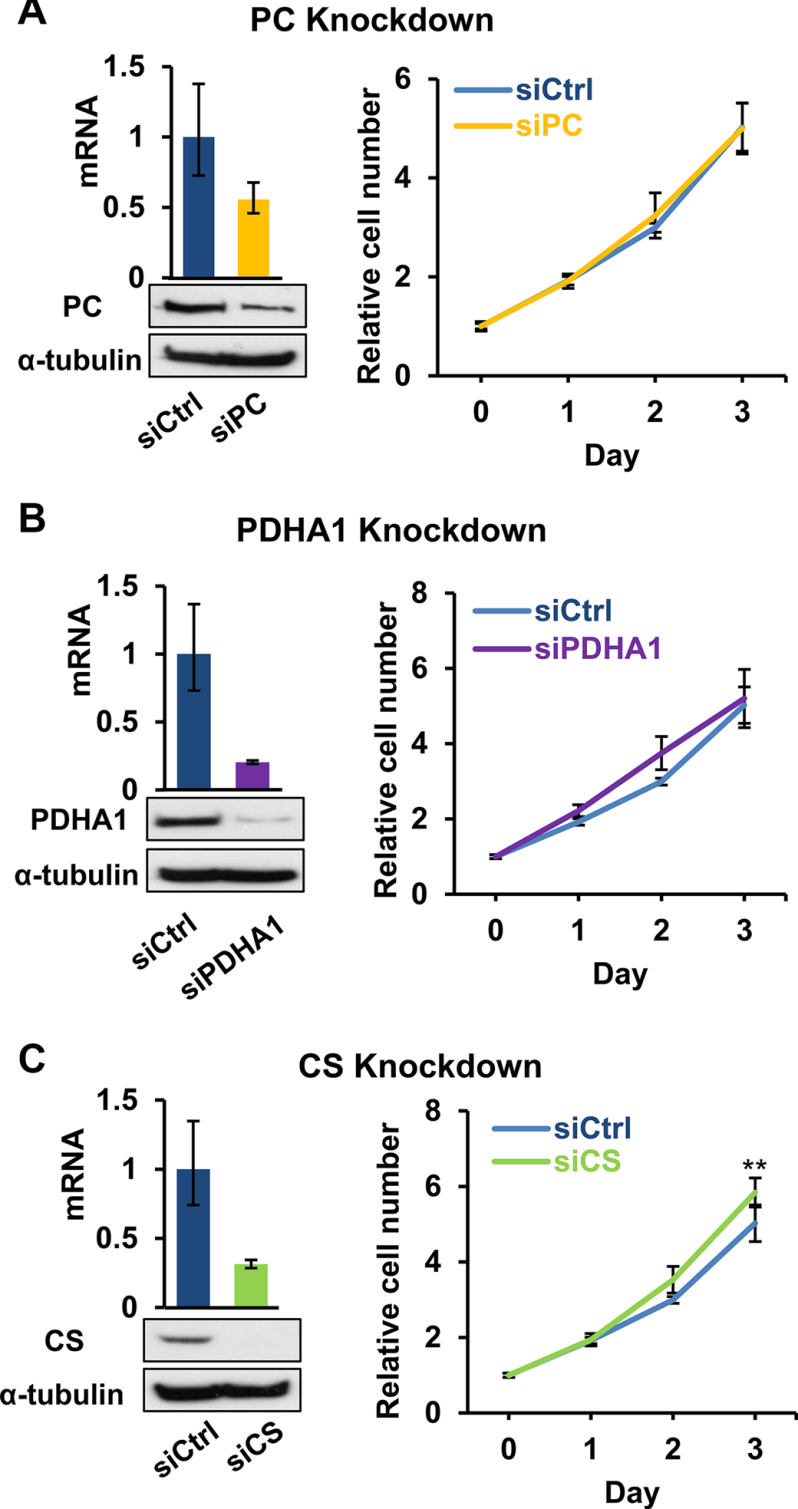
Effects of disruption of pyruvate-based biosynthesis on 143B206 cell proliferation (**A**) PC, (**B**) PDHA1, and (**C**) CS were knocked down in 143B206 cells with siRNA. qRT-PCR (Left top) and immunoblotting (Left bottom) were employed to determine the knockdown efficiency. Proliferation curves (Right) of the control and knockdown 143B206 cells in the presence of exogenous pyruvate were measured. For qRT-PCR, error bars indicate 95% confidence interval of triplicates. For proliferation curves, error bars indicate SD of ≥ 5 replicates. **p < 0.01.

The major biosynthetic role of pyruvate is to serve as a substrate for the biosynthesis of OAA (by pyruvate carboxylase, PC) and acetyl-CoA (by pyruvate dehydrogenase complex, PDC). OAA may serve as the α-keto acid for the synthesis of aspartate, which is needed for protein translation and synthesis of nucleotides as well as asparagine. OAA and acetyl-CoA can be further used in the biosynthesis of citrate by citrate synthase (CS), which is the starting step of Krebs cycle (Figure 2A). Since biosynthesis of membrane phospholipids and cholesterols depends on citrate synthesis, we examined if facilitating the production of OAA and citrate for proliferative biosynthesis underlines the importance of exogenous pyruvate in 143B206. We knocked down PC, PDHA1 (a critical subunit of PDC) and CS in 143B206 cells, and observed knockdown of these enzymes did not impair the proliferation of 143B206 cells in the presence of exogenous pyruvate (Figure 4A–4C). Interestingly, CS knockdown slightly, but significantly promoted 143B206 proliferation (Figure 4C). These data indicate that the critical role of exogenous pyruvate to rescue ETC-defective cells is not to act as a substrate to support biosynthesis of lipids.

### Exogenous pyruvate relieves ATP insufficiency and enhances the proliferation of cells under hypoxic conditions

**Figure 5:**
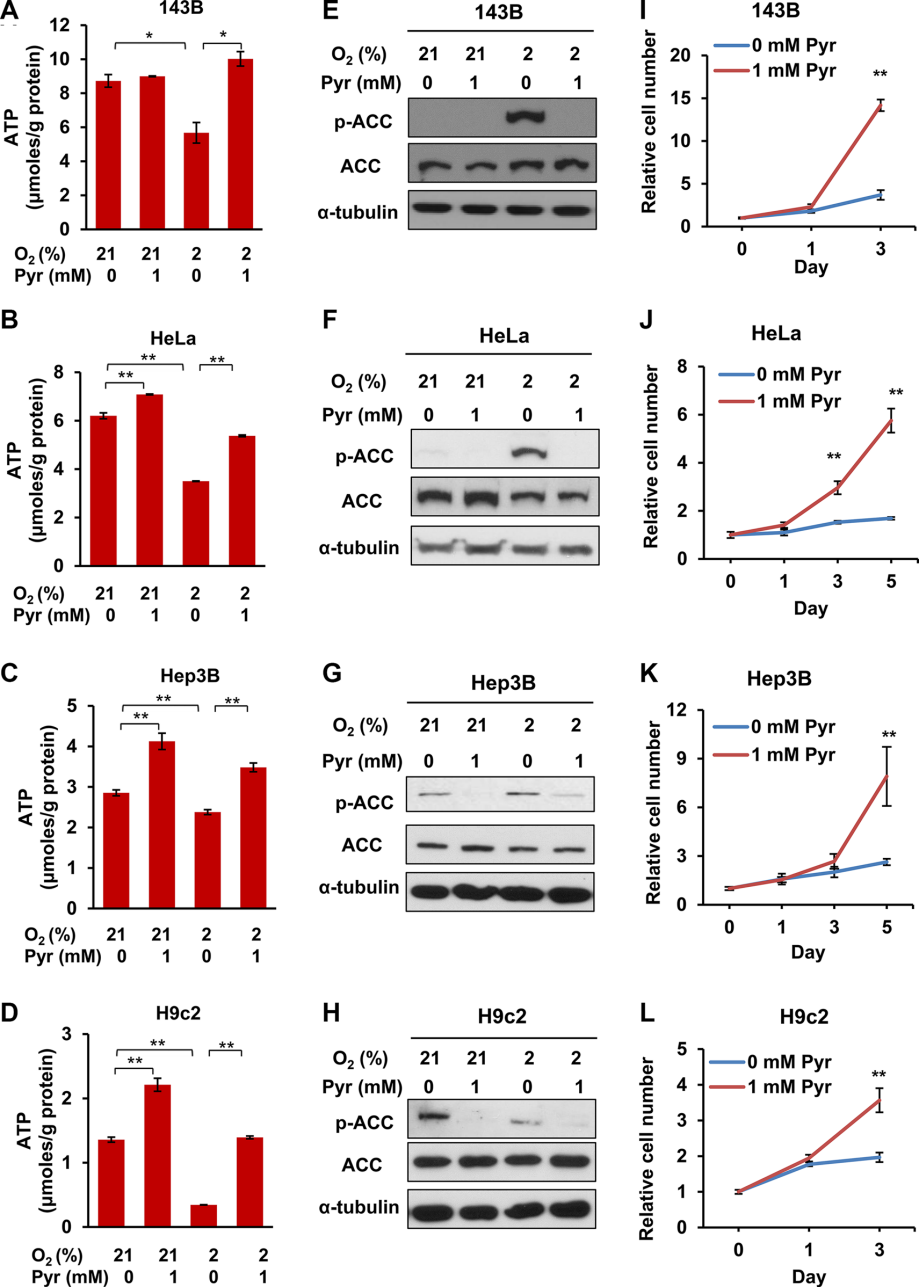
Exogenous pyruvate relieves ATP depletion and sustains the proliferation of both cancer and non-cancer cells under hypoxia (**A**–**D**) 143B, HeLa, Hep3B and H9c2 cells were cultured with indicated conditions for 24 h. Cellular ATP levels were measured and normalized by protein amount. Error bars indicate SD of triplicates. (**E**–**H**) Phosphorylated ACC (Ser79, pACC) and total ACC in whole lysates of 143B, HeLa, Hep3B and H9c2 cells after indicated treatment were analyzed with immunoblotting. Representative results from triplicates are shown. (**I**–**L**) 143B, HeLa, Hep3B and H9c2 cells were cultured in media supplemented with 0 mM or 1 mM pyruvate under 2% oxygen for indicated time, and cell proliferation rates were measured. Representative proliferation curves are shown. Error bars indicate SD of ≥ 5 replicates. *p < 0.05. **p < 0.01.

Based on the above observations, we argue that similar to ETC-deficient cells, cells under severe or prolonged hypoxia may lead to NADH accumulation and NAD^+^ depletion, thus eventually requiring exogenous pyruvate to sustain glycolysis and ATP production. To determine the effect of exogenous pyruvate on intracellular ATP levels and the proliferation rates of prolonged hypoxic cells, we cultured 143B, HeLa, Hep3B and H9c2 cell lines under indicated conditions. We observed that hypoxia significantly decreased ATP levels in all the four cell lines, and exogenous pyruvate acted against hypoxia to fully or partially restore ATP levels (Figure 5A–5D). Interestingly, exogenous pyruvate also increased ATP levels under normoxic conditions in HeLa, Hep3B and H9c2 cells (Figure 5B–5D). Consistently, we observed that AMPK was activated by hypoxia, as indicated by the presence of phosphorylated ACC (Ser79), which was suppressed by exogenous pyruvate (Figure 5E–5H). Phosphorylation of ACC was even observed under 21% O_2_ in Hep3B and H9c2 cells, which may be caused by their relatively low basal levels of ATP (Figure 5G and 5H). When cultured in chronic hypoxic conditions (2% O_2_) the proliferation rates of all cell lines were significantly higher in the presence of exogenous pyruvate (Figure 5I–5L). These data indicate that exogenous pyruvate facilitates the proliferation of both cancer and non-cancer cells under hypoxic conditions by sustaining ATP production. In addition, some cell types, even under normal culture conditions, undergo an energy shortage stress, which may be ameliorated by exogenous pyruvate.

### Exogenous pyruvate alleviates hypoxia-triggered inhibition of mTOR

**Figure 6:**
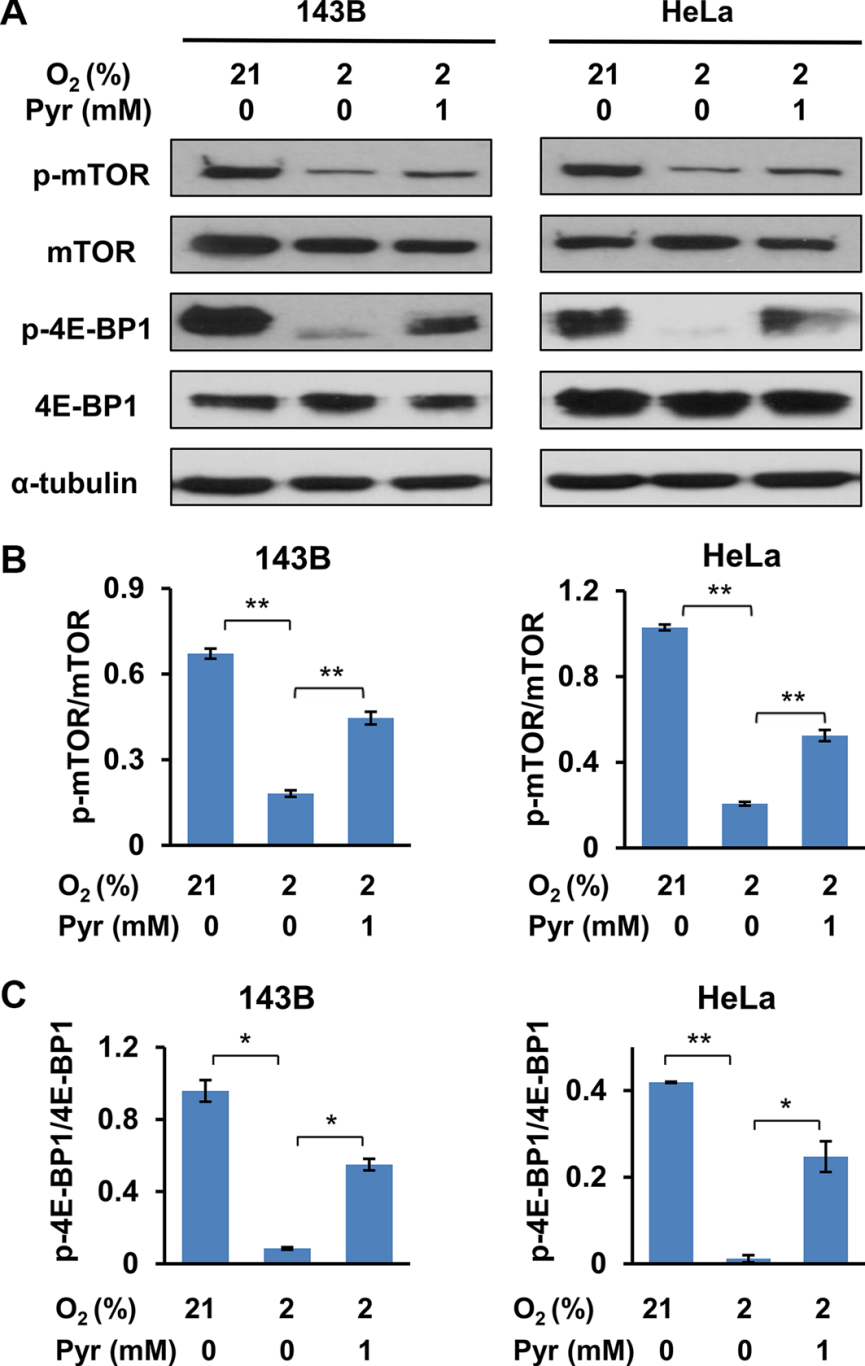
Exogenous pyruvate relieves inhibition of mTOR by hypoxia (**A**) 143B and HeLa cells were treated with indicated conditions for 24 h. Whole cell lysates were prepared, and phosphorylation of mTOR (Ser2448) and 4E-BP1 (Thr37/46) were analyzed with immunoblotting. (**B** and **C**) The specific signals in three repeated Western blots (representative shown in (A)) were quantified. The ratios of phosphorylated mTOR to total mTOR (B) and phosphorylated 4E-BP1 to total 4E-BP1 (C) were shown. Error bars indicate SD from three independent replicates. *p < 0.05. **p < 0.01.

The mTOR signaling pathway is an important nutrient-dependent one that promotes cell growth and proliferation [24]. Sufficient nutrients and energy maintain an active mTOR signaling, which is indicated by mTOR phosphorylation at multiple sites including Thr2446 and Ser2448 [25, 26]. Activated mTOR pathway promotes anabolic processes including protein translation and lipogenesis, and inhibits catabolic processes such as autophagy [27, 28]. Specifically, activated mTOR phosphorylates 4E-BP1 at Thr37/46 to promote protein translation [29]. In addition, it is known that AMPK inhibits mTOR [30]. As exogenous pyruvate facilitates cellular ATP homeostasis under hypoxia, we investigated whether exogenous pyruvate enhances mTOR signaling. Not surprisingly, we observed that phosphorylation of mTOR (Ser2448) and 4E-BP1 (Thr37/46) were inhibited in cells cultured with 2% O_2_, and presence of exogenous pyruvate partially restored the phosphorylation levels of both mTOR and 4E-BP1 (Figure 6). These data indicate that exogenous pyruvate prevents hypoxia-triggered inhibition of mTOR signaling.

### Well-oxygenated cells release pyruvate

**Figure 7:**
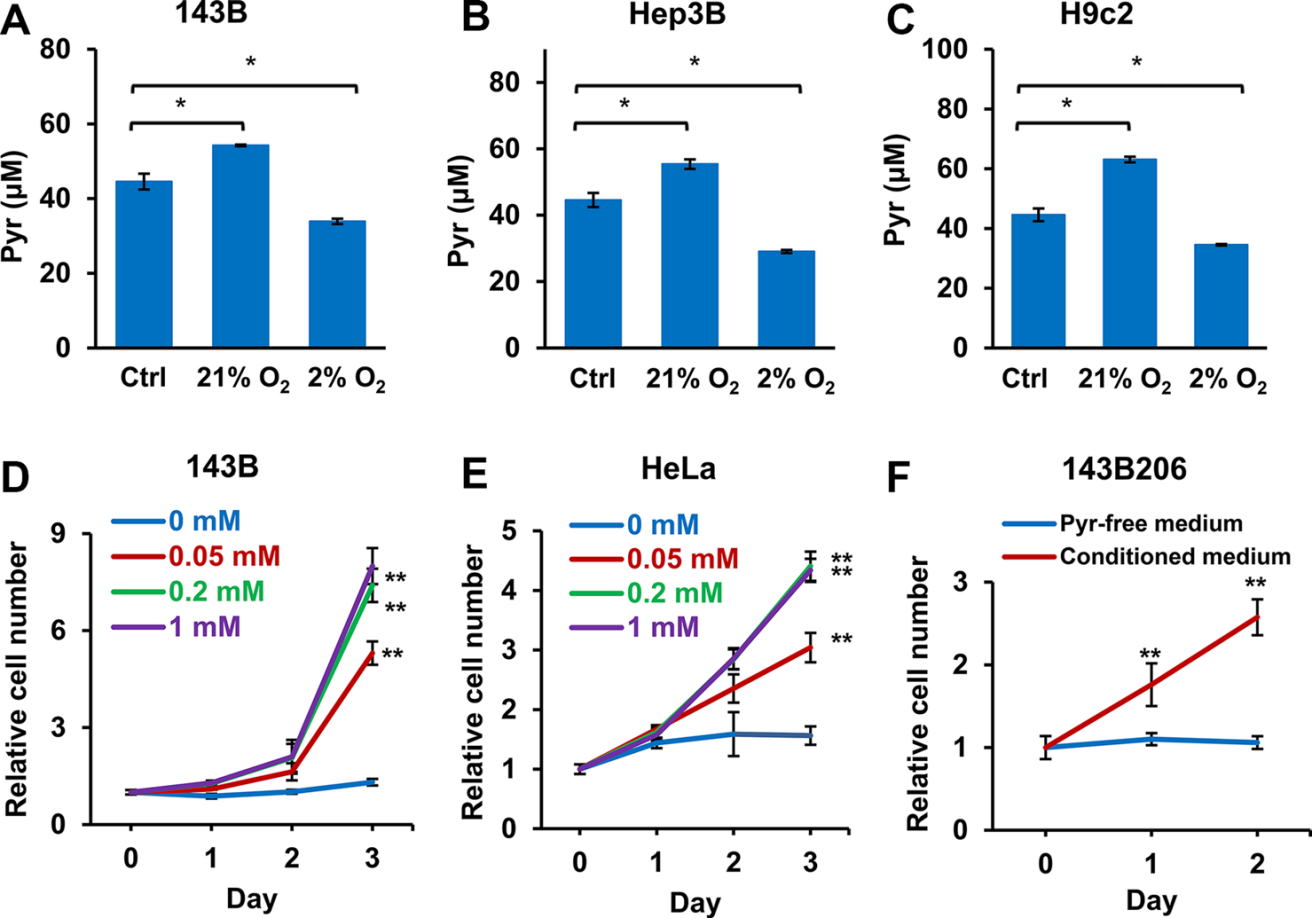
Well-oxygenated cells release pyruvate and hypoxic cells consume pyruvate in the media (**A**) 143B, (**B**) Hep3B, and (**C**) H9c2 cells were seeded in pyruvate-free DMEM supplemented with 10% dialyzed FBS and 50 μM pyruvate and cultured in 21% or 2% oxygen. After 24 h, pyruvate concentrations in culture media were determined. Control groups represented the initial concentration of pyruvate. Error bars indicate SD of triplicates. (**D** and **E**) 143B and HeLa cells were cultured under 2% O_2_ with addition of 0, 0.05, 0.2 or 1 mM pyruvate for 3 days. Cell proliferation rate was measured. (**F**) 143B cells were cultured in pyruvate-free medium under 21% O_2_ for 48 h. The conditioned medium was then collected, supplemented with uridine, and used to culture 143B206 cells. The proliferation rate of 143B206 cells in the conditioned media was measured. Error bars indicate SD of ≥ 5 replicates. *p < 0.05. **p < 0.01.

Next we asked whether well-oxygenated cells release pyruvate, which serves as an *in vivo* source of pyruvate exogenous to hypoxic cells. Normal serum contains about 50 μM pyruvate [31, 32], which was set as the initial concentration of pyruvate in the culture media. We tested 143B, Hep3B and H9c2 cells in media containing 50 μM pyruvate under either normoxic (21% O_2_) or hypoxic (2% O_2_) condition, and then measured the concentrations of pyruvate in the media after 24 h. We found that when cells were cultured in 21% oxygen, the pyruvate levels in the media significantly increased. In contrast, culturing cells in 2% oxygen significantly decreased the pyruvate concentrations in media (Figure 7A–7C), indicating well-oxygenated cells, either tumor or non-tumor cells, may release pyruvate while hypoxic cells consume pyruvate. To determine whether physiological levels of exogenous pyruvate are sufficient to support the proliferation of hypoxic cells, we determined the proliferation rates of hypoxic 143B and HeLa cells cultured in increasing concentrations of pyruvate. As shown in Figure 7D and 7E, 50 μM pyruvate was able to significantly promote the proliferation of hypoxic 143B and HeLa cells; and 200 μM of pyruvate was sufficient to reach the optimal proliferation. Next, we cultured 143B cells in pyruvate-free media for 48 h under 21% O_2,_ and used the conditioned media to culture ETC deficient 143B206 cells. We found that the conditioned media were sufficient to support the proliferation 143B206 cells (Figure 7F). Taken together, these data indicate that the pyruvate released by well-oxygenated cells, may serve as an *in vivo* source of exogenous pyruvate to support the proliferation of hypoxic cells.

## DISCUSSION

In this study, we interrogated the role of exogenous pyruvate in supporting the proliferation of cells defective for oxygen utilization, with an emphasis on the metabolic relevance to hypoxic cells. We showed that exogenous pyruvate significantly alleviated the inhibitory effects of ETC deficiency and hypoxia on both cancer cells (143B206, 143B, HeLa and Hep3B) and non-cancer cells (H9c2, rat cardiomyocytes). Whereas pyruvate may play a role in anaplerosis and participates in multiple biosynthetic pathways, we demonstrate that the rescuing effect of exogenous pyruvate for hypoxic cells is mainly by acting as an oxygen surrogate to accept electrons, thus maintaining cellular NAD^+^ levels to ensure the continuation of glycolysis and ATP production. While the biochemical role of exogenous pyruvate is basically identical to that of endogenous pyruvate derived from intracellular glycolysis, the dependence of exogenous pyruvate for hypoxic cells greatly expands our understanding of the interaction between hypoxic and normoxic cells during the adaptation processes.

Anaerobic glycolysis, described as the Pasteur effect, has been established as a cellular adaptation to hypoxia and aerobic glycolysis, the Warburg effect, has been considered part of the metabolic transformation in cancer cells. In both models, endogenous pyruvate from glycolysis is proposed to support the regeneration of oxidized NAD^+^, facilitating ATP production and other NAD^+^-dependent metabolism. As such, LDHA has been explored as a therapeutic target for cancer therapy [33, 34]. However, in ETC-defective cells, exogenous pyruvate becomes absolutely required for cell survival and proliferation. We note that some metabolites of the glycolytic pathway may be directed to other metabolic pathways such as synthesis of serine and glycerol phosphates, leading to shortage of pyruvate of lactate formation [14]. More importantly, as electron carrier, NAD^+^ not only accepts electrons from the glycolytic pathways, but can also be reduced in other metabolic reactions [35]. It is well known that NAD^+^ is reduced to NADH in a variety of metabolic pathways such as Krebs cycle, oxidation of fatty acids and amino acids. Under conditions of hypoxia, the Krebs cycle is suppressed by HIF-1-mediated upregulation of pyruvate dehydrogenase kinase (PDK1). NADH from limited activity of Krebs cycle and other metabolic reactions may be oxidized by residual amount of molecular oxygen through ETC, hence the role of exogenous pyruvate may not be obvious in relatively mild or transient hypoxia. However, for cells with severe, prolonged hypoxia, or defective ETC when oxygen cannot be used, it becomes clear that endogenous pyruvate is insufficient to oxidize NADH generated from all metabolic processes, leading to severe NAD^+^ depletion, which may eventually inhibit the glycolysis and ATP production. Besides the role in ATP production, NAD^+^ also serves as the substrate of sirtuins and poly (ADP-ribose) polymerases (PARP), important enzymes for cellular signaling pathways and DNA damage repair [36]. Therefore, utilizing exogenous pyruvate to maintain the sufficient level of NAD^+^ is likely to be an important adaptive strategy for cells under severe and prolonged hypoxic conditions.

After entering hypoxic cells, exogenous pyruvate may join the intracellular pyruvate pool, and participate in some important biosynthetic pathways. Particularly, proliferating cells normally have increased demand for the synthesis of serine, glycine, aspartate, NADPH and lipids. Pyruvate, through anaplerotic reactions, may contribute carbon to these synthetic activities. Accordingly, it has been reported that PC is a critical enzyme for some type of cancers, such as non-small cell lung cancer [37]. We observed that substituting pyruvate with pyruvate-derived metabolites or precursors (acetyl-CoA, α-KG, succinate and alanine) cannot rescue the proliferation of 143B206 cells, and the knockdown of enzymes (PC, PDHA1 and CS) catalyzing pyruvate-dependent biosynthesis does not impair pyruvate-mediated rescue of 143B206 cells. This suggests that the biosynthetic role of pyruvate is unlikely the rate-limiting parameter in oxygen-independent cell proliferation. It has been known that tumor cells undergo a dramatic change in cellular metabolism. Warburg effect and increased glutaminolysis are two of the most noticeable metabolic alterations [38, 39]. Through glutaminolysis driven by nitrogen anabolism, glutamine may provide α-KG to replenish the Krebs cycle [40]. Therefore, the carbon flux from glutaminolysis may provide an alternative carbon source to support the proliferative biosynthesis of biomembrane lipids, aspartate and other amino acids [40, 41]. In addition, cells defective in oxygen utilization generally proliferate slower than the parental cells, which may reduce the demand of carbon source for biosynthesis and make it not a rate limiting parameter. These may provide an explanation for why exogenous pyruvate is dispensable for proliferative biosynthesis in cells defective in oxygen utilization.

Interestingly, OAA and aspartate partially rescue the proliferation of 143B206 cells to a certain extent. During the preparation of this report, we noticed that similar observation was reported with different cell models, where the biosynthesis of aspartate was proposed to be the critical role of pyruvate-mediated rescue of ETC-defective cells [42, 43]. In our experimental setting, knockdown of PC did not block 143B206 proliferation rescued by exogenous pyruvate, indicating the pyruvate to OAA pathway is not critical. Alternatively, OAA, as a keto acid, may similarly act as an electron acceptor in the reaction catalyzed by malate dehydrogenase (MDH1), and aspartate may rescue 143B206 proliferation by being catabolized to OAA, a reaction catalyzed by cytosolic aspartate aminotransferase (GOT1) (Supplementary Figure S2B and S2C). Consistent with this explanation, inhibiting citrate synthesis by CS knockdown not only relieves the NADH accumulation, but also conserves OAA. While theoretically, most keto-acids may serve as electron acceptors to regenerate NAD^+^, we note that pyruvate shows significantly better rescuing effects than OAA. The underlying biochemical mechanisms remain unknown, but a plausible explanation is the thermodynamic and enzymatic difference among these reactions.

**Figure 8:**
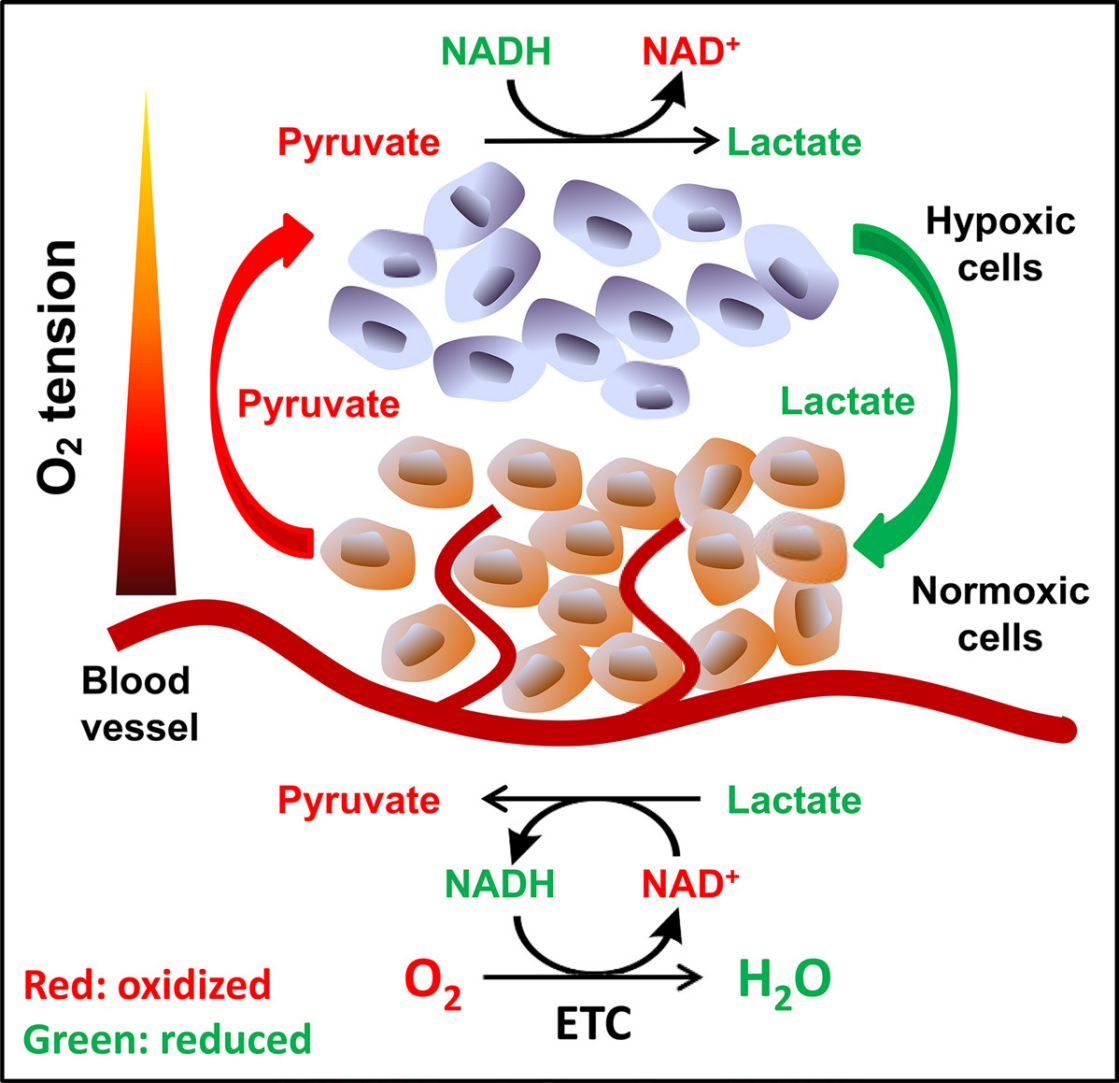
Proposed model of pyruvate cycle Tissue oxygen concentration decreases as the distance between cells and blood vessels increases. Cells in well-oxygenated areas mainly use ETC to regenerate NAD^+^; and a portion of pyruvate generated from glycolysis is released to the circulation, which may diffuse to hypoxic region. Hypoxic cells uptake exogenous pyruvate and use it as oxygen surrogate to maintain NAD^+^ availability, avoiding NAD^+^ depletion and eventual inhibition of glycolysis. In turn, the lactate released from hypoxic cells may be up-taken by the well-oxygenated cells and be oxidized to pyruvate.

Data from cell culture studies clearly indicate that exogenous pyruvate enhances cell viability and supports oxygen-independent proliferation of ETC-defective cells. Within an *in vivo* environment, the source of pyruvate exogenous to solid tumor cells is an interesting question. We have shown that well-oxygenated cells release pyruvate into culture media. It has been well-known that hypoxic tumor cells export lactate and well-oxygenated tumor cells uptake lactate as substrate for metabolism [44, 45]. Taking these facts together, we propose that adjacent well-oxygenated cells, including normal cells, tumor cells or stromal cells, may release pyruvate, while hypoxic cells uptake and use the exogenous pyruvate as an oxygen surrogate to maintain the intracellular NAD^+^ levels to survive and proliferate (Figure 8).

It is known that cell surface monocarboxylate transporters (MCT1, 2, and 4) are upregulated by hypoxia [46], which may facilitate hypoxic cells to uptake exogenous pyruvate. In fact, recent studies have showed the efficacy of MCT inhibitors in killing cancer cells [47, 48]. How the secretion of pyruvate by non-hypoxic cells is regulated, and particularly, whether hypoxic or cancer cells positively regulate the pyruvate secretion from adjacent, well-oxygenated cells remains to be investigated. Intracellularly, mitochondrial membrane pyruvate transporters were discovered recently [49, 50]. Whether these transporters play a role in the rescuing effects of exogenous pyruvate by increasing mitochondrial uptake of pyruvate also remains to be studied. Nevertheless, limiting the utilization of exogenous pyruvate may be a promising approach to treat hypoxic cancer cells, which are often resistant to chemotherapy and radiation.

The best known cellular response to hypoxia includes stabilization of HIF-1 and activation of AMPK. Increased AMP concentration as a result of ATP shortage activates AMPK [51]. The AMPK pathway inhibits energy-consuming processes such as fatty acid synthesis and protein synthesis, and upregulates energy generation pathways to increase ATP levels [51]. Activated AMPK also facilitates the activation of HIF-1 by nuclear exporting HDAC5, which promotes HIF-1α maturation and nuclear localization [52]. In addition to HIF-1 and AMPK signaling pathways, hypoxia-caused ATP shortage and ROS accumulation may trigger other cellular adaptive responses including the endoplasmic reticulum (ER) stress response. ER stress response represents an important adaptive mechanism for cell adaptation to stresses including hypoxia [53]. Severe and prolonged hypoxia may overwhelm cells' capacity to adapt, and eventually leads to apoptosis via the intrinsic pathway [54]. In this study we have shown that exogenous pyruvate relieves hypoxia-triggered AMPK activation. How exogenous pyruvate may modulate ER stress response and apoptosis in hypoxic cells remains to be investigated.

In summary, this study provides evidence to support that exogenous pyruvate is required to support oxygen-independent survival and proliferation of cancer and non-cancer cells under prolonged hypoxic conditions. The protective effect is dependent on the ability of pyruvate to act as an oxygen surrogate to accept electrons, hence maintaining the intracellular NAD^+^ levels to ensure ATP production. The biosynthetic role of exogenous pyruvate is dispensable. These findings expand our current understanding of the Pasteur and Warburg effects, provide new insight into the interaction between cancer cells and adjacent cells, and pave a way towards the development of potential new treatment for hypoxic cancers, based upon targeting the utilization of exogenous pyruvate, either alone or as a sensitizer for chemotherapy or radiation.

## MATERIALS AND METHODS

### Cell culture

HeLa and Hep3B were purchased from ATCC (Chicago, IL). H9c2 was a kind gift from Dr. P. Lelkes (Temple University, Philadelphia, PA). 143B and 143B206 were kind gifts from Dr. M. King (Thomas Jefferson University, Philadelphia, PA). All cell lines were cultured in Dulbecco's modified Eagle's medium (DMEM) containing 25 mM glucose, 1 mM pyruvate and 4 mM glutamine supplemented with 10% fetal bovine serum (FBS), unless otherwise indicated. The culture medium for 143B206 cells was further supplemented with 50 μg/mL uridine. All cells were cultured in humidified 5% CO_2_/95% air atmosphere at 37°C. For pyruvate-deprivation study, cells were cultured in pyruvate-free DMEM with 25 mM glucose, 4 mM glutamine and 10% dialyzed FBS. Hypoxia treatement was carried out by culturing cells directly in a hypoxic Workstation (Invivo_2_ 300, Ruskinn Technology, Sanford, ME) equipped with a programmable controller of humidity, temperature and premixed gas (2% O_2_, 5% CO_2_, balanced with N_2_).

### Cell proliferation assay

Cell proliferation rates were determined by CyQUANT^®^ NF Cell Proliferation Assay Kit (Invitrogen, Carlsbad, CA) according to the manufacturer's protocol. In brief, cells were plated at density of 100–500 cells per well in a 96-well microplate. Cell numbers in wells were measured every one or two days. Culture medium was removed gently, 50 μL of CyQUANT^®^ NF dye solution (which exhibits strong fluorescence enhancement after binding with DNA) was added to the well and then the microplate was covered and incubated at 37°C for 30 min. The fluorescence intensity was measured using a fluorescence microplate reader with excitation at 485 nm and emission detection at 530 nm. As DNA content is closely proportional to cell number, the assay is designed to produce a linear analytical response from at least 100–20,000 cells per well in a 96-well microplate. The relative cell number stands for the ratio of cell number at indicated time to the starting cell number at the time of treatment.

### Microscopy analysis of living cell morphology

143B and 143B206 cells were seeded in 6-well culture plates and cultured in the medium with 1 mM pyruvate or not for 48 h. Images of the living cells were photographed under inverted Axioplan phase-contrast microscope (Zeiss, Thornwood, NY) at 400 × magnification, and analyzed with the software Slidebook6 (3i, Denver, CO).

### Extracellular acidification rate (ECAR)

ECAR was measured using Seahorse XF24 extracellular Flux analyzer (Seahorse Bioscience, North Billerica, MA) following the manufacturer's instructions. In brief, 143B and 143B206 cells were seeded at a density of 2 × 10^4^ and 3 × 10^4^ cells/well respectively into Seahorse 24-well microplates and allowed to grow for 18 h. Thirty minutes before the assay, the culture medium was replaced with unbuffered XF assay medium (pH7.4) supplemented with 25 mM glucose and 1 mM or 0 mM pyruvate, and incubated at 37°C for 30 minutes for stabilization of pH and temperature. After all the measurements were completed, cells were treated with trypsin and counted. These cell counts were used to normalize ECAR.

### ATP concentration assay

The ATP concentrations in cells were determined with ATP Determination Kit (Thermo Fisher Scientific) based on the manufacturer's protocol and literature [55]. Briefly, cells were plated in 60 cm culture dishes and cultured overnight. Then the cells were treated with indicated conditions. After 24 hours of treatment cells were lysed in 1% NP40 (plus protease inhibitors) and centrifuged at 14,000 rpm for 15 min. The supernatant was collected as cell lysates for ATP assay. Protein concentrations in lysates were measured with Bio-Rad Protein Assay Dye Reagent Concentrate. ATP levels were normalized by protein concentrations.

### NAD^+^ and NADH assay

Intracellular NAD^+^ and NADH levels were determined with the Fluoro NAD/NADH^™^ Detection Kit (Cell Technology, Mountain View, CA) according to the manufacturer's protocol. Briefly, 143B and 143B206 cells were seeded at a density of 2 × 10^6^ and 3 × 10^6^ cells into a 10 cm culture dish respectively and cultured under 21% or 2% oxygen with 0 mM or 1 mM pyruvate for 24 h. Cells were harvested and lysed with provided lysis buffer. The lysates were then centrifuged at 8,000 rpm for 5 min at 4°C. The supernatant was retrieved for subsequent NAD^+^ and NDAH measurement with excitation 530-570 nm/emission 590-600 nm fluorescent assay. The NAD^+^/NADH ratio was then calculated. A parallel culture dish of both cells were cultured under the same condition and then counted for normalization of NAD^+^ levels.

### Knockdown with siRNA

Knockdown of pyruvate carboxylase (PC) (EC number: 6.4.1.1), pyruvate dehydrogenase (lipoamide) alpha 1 (PDHA1) (EC number: 1.2.4.1), citrate synthase (CS) (EC number: 2.3.3.16), and lactate dehydrogenase A (LDHA) (EC number: 1.1.1.27) were performed using the specific Invitrogen *Silencer*^®^ Select siRNAs (PC siRNA ID: s10089, PDHA1 siRNA ID: s10245, CS siRNA ID: s3583, LDHA siRNA ID: s351). *Silencer*^®^ Select Negative Control siRNA served as negative control. Lipofectamine^TM^ 2000 Transfection Reagent (Invitrogen) was used for the transfection of siRNAs. Briefly, cells were seeded into a 60-mm culture dish and were allowed to achieve 95% confluence. 200 pmol siRNA and 6 μL Lipofectamine 2000 Transfection Reagent were incubated in 0.5 mL Gibco Opti-MEM^®^ I Reduced-Serum Medium (Thermo Fisher Scientific) for 5 min, respectively, and then mixed and incubated for 20 min. Cells were transfected with the mixed medium. 6 h after transfection, cells were seeded in 96-well microplates for cell proliferation assay, and in 60 mm culture dishes for RNA and protein extraction.

### Quantitative real-time PCR (qRT-PCR)

Total RNA was extracted with Qiagen RNeasy kit (Invitrogen). cDNA was synthesized using SuperScript II Reverse Transcriptase (Invitrogen), and then used for quantitative analysis with specific TaqMan probes for PC (Hs00559398_m1), PDHA1 (Hs01049345_g1), CS (Hs02574374_s1) and β-actin (Hs01060665_g1) in StepOnePlus Real-Time PCR System (Applied Biosystems, Foster City, CA). cDNA was amplified in 20 μL reactions containing 1 μL of probe and 10 μL of 2 × Taqman^®^ Gene Expression Master Mix (Applied Biosystems). The procedure applied was 50°C for 2 min and 95°C for 10 min at Stage 1, 95°C for 15 s and 60°C for 1 min for 40 cycles at Stage 2. Data were quantitatively analyzed by the software StepOne^™^ v2.1 (Applied Biosystems) with comparative C_T_ method. β-actin was used as endogenous control. All genes were analyzed in triplicates.

### Immunoblotting

Cells were lysed in RIPA buffer (50 mM Tris-HCl, pH 7.4, 150 mM NaCl, 0.5% Sodium deoxycholate, 0.1% SDS and 1% NP-40), homogenized and centrifuged at 14,000 rpm for 15 min. The supernatant was collected as whole cell lysates. Protein concentrations in the lysates were measured with Bio-Rad Protein Assay Dye Reagent Concentrate (BIO-RAD, Hercules, CA). 30 μg of proteins were separated by 8% SDS-PAGE, transferred to PVDF membrane (BIO-RAD), and probed with specific primary antibodies including rabbit anti-phospho-acetyl-CoA carboxylase (Ser79) (Catalog # 11818S), rabbit anti-acetyl-CoA carboxylase (Catalog # 3662S), rabbit anti-LDHA (Catalog # 3582S), rabbit anti-phospho-mTOR (Ser2448) (Catalog # 5536S), rabbit anti-mTOR (Catalog # 2983S), rabbit anti-phospho-4E-BP1 (Thr37/46) (Catalog # 2855S), and rabbit anti-4E-BP1 (Catalog # 9452S) from Cell Signaling, mouse anti-pyruvate carboxylase (Catalog # sc-271493), mouse anti-PDHA1 (Catalog # sc-377092), mouse anti-citrate synthase (Catalog # sc-390693) from Santa Cruz, and mouse anti-α-tubulin (Catalog # T9026, Sigma). The primary antibodies were detected with appropriate secondary antibody including horseradish peroxidase-conjugated goat anti-mouse (catalog #A4416) or goat anti-rabbit (catalog #A6154) from Sigma and developed with SuperSignal^®^ West Pico Chemiluminescent Substrate (Thermo Fisher Scientific). The detected bands were quantified with ImageJ (NIH, Bethesda, MD).

### Pyruvate concentration assay

Cells were cultured in 6-well plates with 2 mL culture media in each well. Culture medium was replaced with pyruvate-free DMEM supplemented with 10% dialyzed FBS and 50 μM pyruvate, and then the cells were cultured in 2% O_2_ at 37°C for 24 h. An aliquot of incubation medium was subsequently collected for measurement of pyruvate concentration with the Pyruvate Assay Kit (Eton Bioscience, San Diego, CA) according to the manufacturer's instruction.

### Data analysis

Data in the Figures are presented as mean values ± SD. Student's *t*-test and multivariate analysis of variance were used for statistical analysis with GraphPad Prism 6.0 (GraphPad Software, San Diego, CA). Differences in all the tests were considered as statistically significant at *p* value < 0.05.

## SUPPLEMENTARY MATERIALS


