# Supplementary material for: Exogenous pyruvate facilitates cancer cell adaptation to hypoxia by serving as an oxygen surrogate
Source: Oncotarget. 2016 Jun 21;7(30):47494–510. doi: 10.18632/oncotarget.10202 (PMC5216956; doi:10.18632/oncotarget.10202)
Supplement: Supplementary file 1 [file oncotarget-07-47494-s001.pdf]

Exogenous pyruvate facilitates cancer cell adaptation to hypoxia by serving as an oxygen surrogate

Supplementary Materials

DISCUSSION

Potential role of exogenous pyruvate in quenching hypoxia-triggered superoxide

Low levels of ROS may contribute to the activation of signaling pathways and the promotion of cellular proliferation, but excessive cellular ROS may damage DNA and proteins, and induce apoptosis [1]. It has been reported that acute and severe hypoxia (< 1% O_2) induces a burst of superoxide which mainly results from impaired ETC [2]. Pyruvate has been reported as a protective anti-oxidant in non-transformed cells [3]. To evaluate the role of exogenous pyruvate as an ROS scavenger in hypoxic cells, we also measured superoxide levels in 143B, 143B206 and Hep3B cells under hypoxia in the presence or absence of exogenous pyruvate. Our results showed that prolonged hypoxia did not increase the levels of superoxide under our experimental conditions (Supplementary Figure S3). In particular, the cellular superoxide levels in 143B206 cells were hardly detectable, consistent with the ETC defective nature of the cells (Supplementary Figure S3A). In 143B cells, hypoxia significantly decreased superoxide levels, and exogenous pyruvate actually increased the generation of superoxide (Supplementary Figure 3B). In Hep3B cells no obvious increase of superoxide generation was triggered by hypoxia; on contrary, addition of exogenous pyruvate increased superoxide levels (Supplementary Figure S3C). These data indicate that the proliferation-promoting effect of exogenous pyruvate on hypoxic cells is unlikely linked to its role in quenching superoxide.

How does exogenous pyruvate reach cells located in lesions with poor circulation?

Except for some rare situations such as high altitude, hypoxia is commonly associated with poor circulation. How does exogenous pyruvate reach cells located in lesions with poor circulation? Based on Henry's gas solubility law, oxygen solubility is dependent on solubility coefficient and oxygen partial pressure. Oxygen solubility coefficient in plasma is $1.39 \cdot 10^{-3}$ $mM \cdot mmHg^{-1}$ [4]. Considering the average oxygen partial pressure in tumor tissues is around 10 mm Hg or less [5, 6], we can calculate that oxygen concentration in tumor tissue plasma is around 13.9 µM. Pyruvate, as an α-keto acid, has a solubility as high as 900 mM in plasma. Also, pyruvate can be diffused in the form of ion without the limitation of partial pressure, ensuring the availability of pyruvate in solid tumors and ischemic lesions.

METHODS

Oxygen consumption rate (OCR)

OCR was measured using Seahorse XF24 extracellular Flux analyzer (Seahorse Bioscience, North Billerica, MA) following the manufacturer's instructions. In brief, 143B and 143B206 cells were seeded at a density of 2×10^4 and 3×10^4 cells/well respectively into Seahorse 24-well microplates and allowed to grow for 18 h. Thirty minutes before the assay, the culture medium was replaced with unbuffered XF assay medium (pH7.4) supplemented with 25 mM glucose and 1 mM or 0 mM pyruvate, and incubated at 37°C for 30 minutes for stabilization of pH and temperature. Mitochondrial respiration inhibitors a. 1 µM oligomycin, b. 300 nM carbonyl cyanide-4-(trifluoromethoxy)phenylhydrazone and c. 100 nM antimycin and retenone were used to test the ETC function shown as test groups. After all the measurements were completed, cells were treated with trypsin and counted. These cell counts were used to normalize OCR.

Superoxide detection

Total ROS/Superoxide Detection kit (Enzo Life Sciences, Farmingdale, NY) was used according to the manufacturer's instructions to detect the cellular superoxide levels under the conditions specified in experiments. Cells were cultured in Falcon 8-chamber slide (Fisher Scientific) under certain conditions for 24 h and then incubated with Superoxide Detection Solution for 1 hour. After washing with PBS buffer twice, cells were mounted and observed with Olympus FV1000 inverted confocal microscope with (550/620 nm) filter at 400 × magnification and photographed with FV10-ASW (Olympus, Shinjuku, Tokyo, Japan). Red signal indicates the presence of superoxide. Average fluorescence intensity per cell was quantified with ImageJ (NIH).

REFERENCES

1. Le Belle JE, Orozco NM, Paucar AA, Saxe JP, Mottahedeh J, Pyle AD, Wu H, Kornblum HI. Proliferative Neural Stem Cells Have High Endogenous ROS Levels that Regulate Self-Renewal and Neurogenesis in a PI3K/Akt-Dependant Manner. Cell Stem Cell. 2011; 8:59–71.
2. Giordano FJ. Oxygen, oxidative stress, hypoxia, and heart failure. J Clin Invest. 2005; 115:500–508.
3. Hinoi E, Takarada T, Tsuchihashi Y, Fujimori S, Moriguchi N, Wang L, Uno K, Yoneda Y. A molecular mechanism of pyruvate protection against cytotoxicity of reactive oxygen species in osteoblasts. Mol Pharmacol. 2006; 70:925–935.
4. Valabregue R, Aubert A, Burger J, Bittoun T, Costalat R. Relation between cerebral blood flow and metabolism explained by a model of oxygen exchange. J Cerebr Blood F Met. 2003; 23:536–545.
5. Lawrentschuk N, Poon AMT, Foo SS, Putra LGJ, Murone C, Davis ID, Bolton DM, Scott AM. Assessing regional hypoxia in human renal tumours using 18F-fluoromisonidazole positron emission tomography. Bju Int. 2005; 96:540–546.
6. Hockel M, Vaupel P. Tumor hypoxia: Definitions and current clinical, biologic, and molecular aspects. J Natl Cancer I. 2001; 93:266–276.

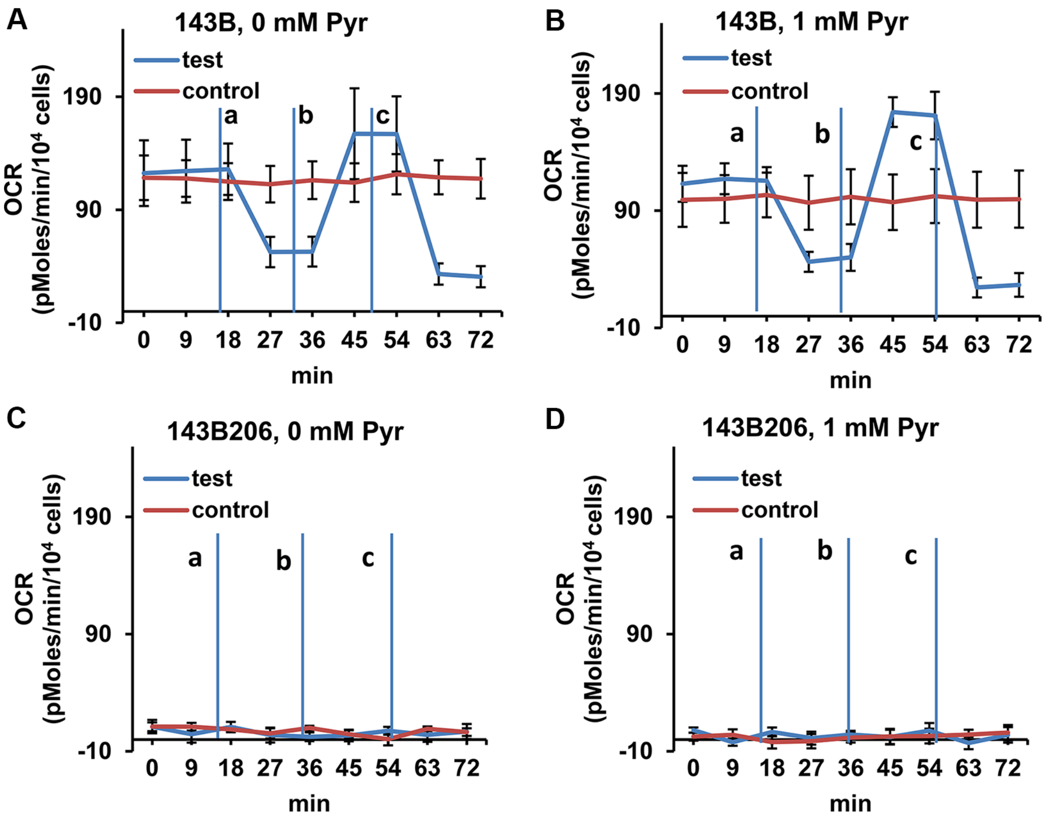

Supplementary Figure S1: Determining the OCR of 143B and 143B206 cells. (**A–D**) Oxygen consumption rate (OCR) of 143B and 143B206 cells were measured with Seahorse metabolic analyzer. Mitochondrial respiration inhibitors a. 1 μM oligomycin, b. 300 nM carbonyl cyanide-4-(trifluoromethoxy) phenylhydrazone and c. 100 nM antimycin and retenone were used to test the ETC function shown as test groups. Control groups were 143B and 143B206 cells without injections of these inhibitors. 143B cells showed typical OCR profiles similar to other cancer cells, while OCR in 143B206 cells was close to 0 pmoles/min, which was not affected by the presence of exogenous pyruvate or the mitochondrial respiration inhibitors, confirming 143B206 cells have no ETC consumption of oxygen. Trace amount, basal level oxygen consumption may be due to non-ETC redox. Error bars indicate SD of ≥ 5 replicates.

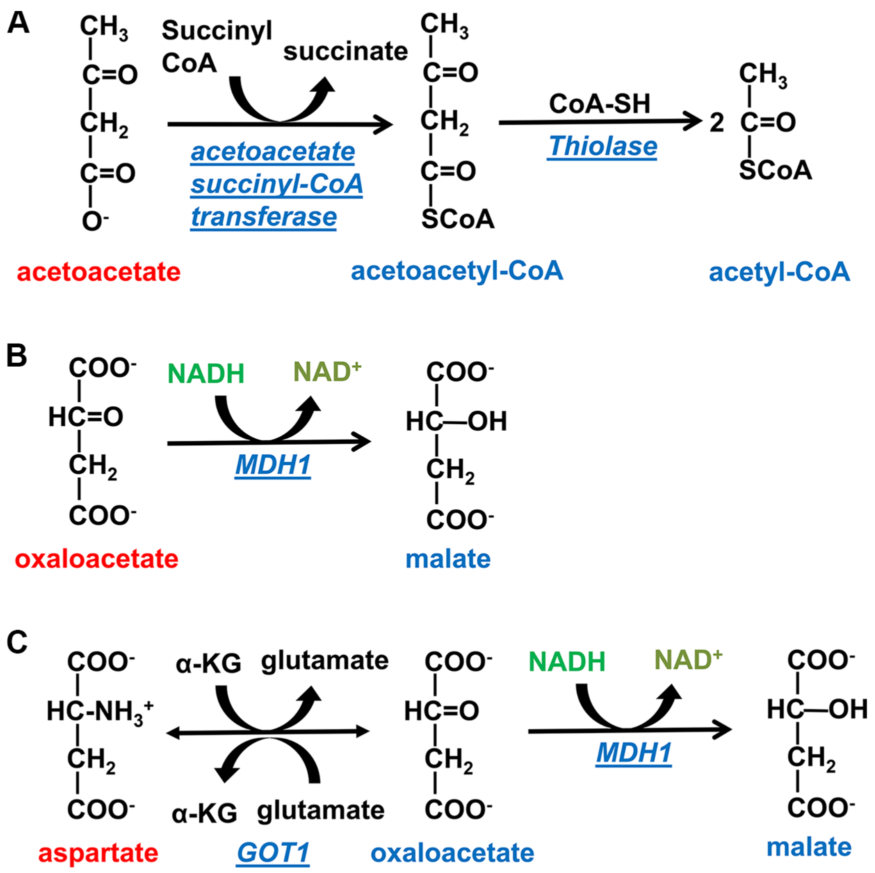

Supplementary Figure S2: Intracellular metabolic pathways of acetoacetate, oxaloacetate and aspartate. (**A**) Intracellular metabolism of acetoacetate. Acetoacetate can be converted to acetoacetyl-CoA by cytosolic acetoacetate succinyl-CoA transferase, and then cleaved to two molecules of acetyl-CoA by thiolase. (**B**) Proposed pathways for OAA-mediated rescue of 143B206 cells. OAA is converted to malate by cytosolic malate dehydrogenase (MDH1) to recycle NAD^+. (**C**) Proposed pathways for aspartate-mediated rescue of 143B206 cells. Aspartate can be deaminated to OAA by cytosolic aspartate aminotransferase (GOT1), which is further reduced to malate while oxidizing NADH to NAD^+.

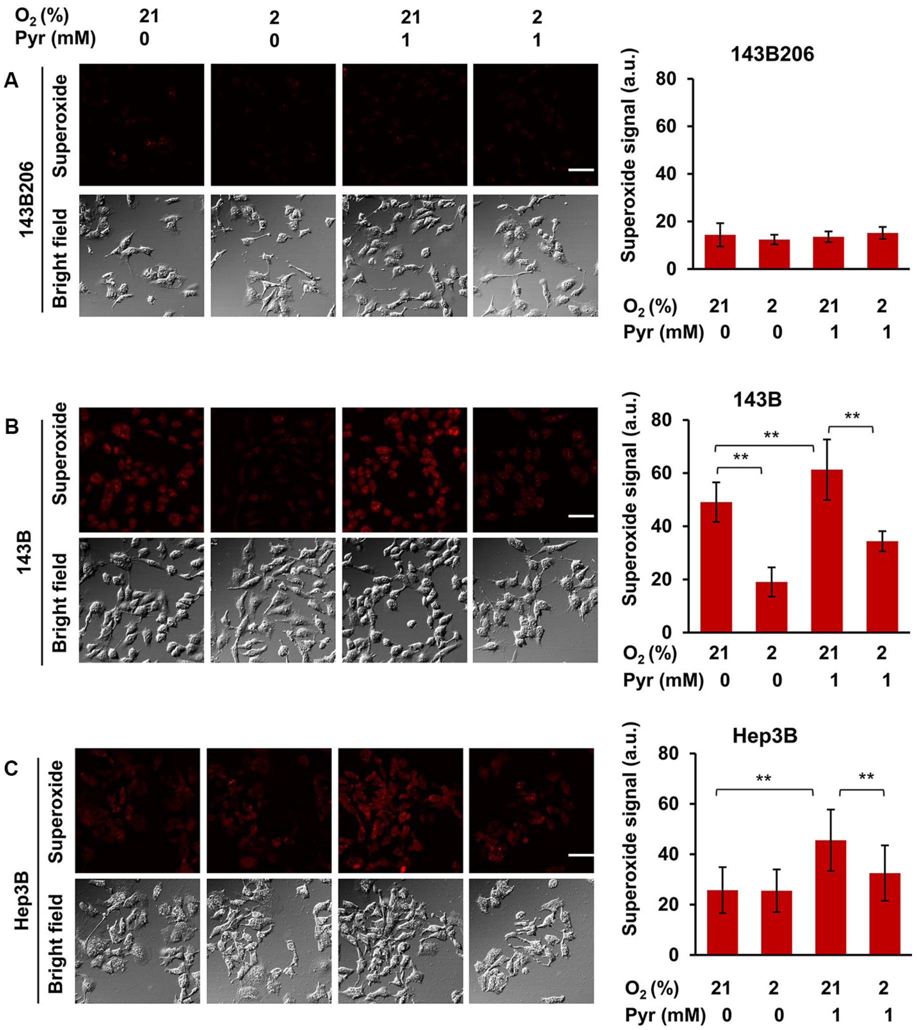

Supplementary Figure S3: Exogenous pyruvate does not decrease the intracellular superoxide level. (**A**) 143B206, (**B**) 143B and (**C**) Hep3B cells were cultured with 21% or 2% oxygen in the presence or absence of 1 mM pyruvate. After 24 h, superoxide levels were examined with superoxide detection assay, and the signals were observed under confocal microscopy. Superoxide-positive pixels per cell were quantified and shown. Error bars indicate SD. Bars represent 100 µm. $^{**}p < 0.01$. a.u., arbitrary units.
